# Bioimaging: An Useful Tool to Monitor Differentiation of Human Embryonic Stem Cells into Chondrocytes

**DOI:** 10.1007/s10439-015-1443-z

**Published:** 2015-09-09

**Authors:** Wiktoria M. Suchorska, Michał S. Lach, Magdalena Richter, Jacek Kaczmarczyk, Tomasz Trzeciak

**Affiliations:** Radiobiology Lab, Greater Poland Cancer Centre, Garbary 15th Street, 61-866 Poznan, Poland; Postgraduate School of Molecular Medicine, Warsaw University of Medical Sciences, Warsaw, Poland; Department of Orthopaedics and Traumatology, Poznan University of Medical Sciences, Poznan, Poland

**Keywords:** Immunofluorescence, Chondrogenesis, Regenerative medicine, Osteoarthritis, Cartilage injuries

## Abstract

To improve the recovery of damaged cartilage tissue, pluripotent stem cell-based therapies are being intensively explored. A number of techniques exist that enable monitoring of stem cell differentiation, including immunofluorescence staining. This simple and fast method enables changes to be observed during the differentiation process. Here, two protocols for the differentiation of human embryonic stem cells into chondrocytes were used (monolayer cell culture and embryoid body formation). Cells were labeled for markers expressed during the differentiation process at different time points (pluripotent: NANOG, SOX2, OCT3/4, E-cadherin; prochondrogenic: SOX6, SOX9, Collagen type II; extracellular matrix components: chondroitin sulfate, heparan sulfate; beta-catenin, CXCR4, and Brachyury). Comparison of the signal intensity of differentiated cells to control cell populations (articular cartilage chondrocytes and human embryonic stem cells) showed decreased signal intensities of pluripotent markers, E-cadherin and beta-catenin. Increased signal intensities of prochondrogenic markers and extracellular matrix components were observed. The changes during chondrogenic differentiation monitored by evaluation of pluripotent and chondrogenic markers signal intensity were described. The changes were similar to several studies over chondrogenesis. These results were confirmed by semi-quantitative analysis of IF signals. In this research we indicate a bioimaging as a useful tool to monitor and semi-quantify the IF pictures during the differentiation of hES into chondrocyte-like.

**Electronic supplementary material:**

The online version of this article (doi:10.1007/s10439-015-1443-z) contains supplementary material, which is available to authorized users.

## Introduction

Articular cartilage is an avascular tissue composed of extracellular matrix (ECM) and cartilage cells (chondrocytes). The main components of ECM are collagens type II, IX, and X, and proteoglycans such as heparan sulfate, keratan sulfate, chondroitin sulfate, and hyaluronic acid. Cartilage performs many important biomechanical functions within the joint, such as absorption of mechanical stress and reduction of friction of the articular surface, enabling painless motion.^5^ As a result of mechanical injuries or catabolic processes (degradation of ECM components by metalloproteinases), chondrocytes become hyper-activated, which triggers hypertrophy and the mineralisation process. The limited regeneration capacity of damaged tissue often leads to osteoarthritis. One procedure for cartilage lesion repair is filling in of damage with autologous chondrocytes propagated *in vitro* (autologous chondrocyte implantation).^23^ However, down-regulation of genes responsible for the production of specific ECM components, for example type II collagen, alongside limited proliferation of primary chondrocytes and their hypertrophic phenotype development during cell culture, may result in loss of tissue functionality after transplantation.^11^,^56^,^57^ One predominant aim of studies in the area of cartilage repair is to obtain fully functional tissue with the properties of native cartilage.

Pluripotent stem cells, including human embryonic stem cells (hESCs), induced-pluripotent stem cells (iPSCs), and multipotent mesenchymal stem cells (MSCs), are undergoing intensive investigation as potential candidates for the treatment of numerous degenerative diseases.^17^,^55^ Because chondrocytes develop from the mesoderm, MSCs are likely to be a suitable cell source for cartilage regeneration. However, obtaining MSCs requires an invasive bone marrow biopsy. Another disadvantage of applying MSCs is their low contribution to the population of bone marrow cells (below 0.001%).^42^ However, many studies involved in characterization of cells population have indicated an alternative source of MSCs. They could be found in adipose tissue, umbilical cord blood, synovium, dental pulp, placenta, *etc*.^13^,^33^ Owing to ethical concerns for the application of hESCs, iPSCs are a good candidate for stem cell-based therapies. To the date, intensively researched reprogramming protocols have led to readily obtaining iPS cells, which are considered safe in clinical practice applications. They give a tremendous possibilities due to ability to differentiate into most cells of organism and unlimited self-renewal capacity. Studies over differentiation of hESCs and iPSC allow to expand the knowledge of developmental biology by tracking of variable molecular pathways, which are activated during differentiation into various progenitors. Additionally, they seems to be suitable source of cells for drugs toxicity assays and disease models.^10^,^25^,^50^,^52^

One major problem in the direct differentiation of pluripotent cells into progenitor cells is that these protocols result in obtaining a heterogeneous population of differentiated cells. A second problem is in achieving efficient cell differentiation on a large scale. Minor modifications of the protocols are now required to obtain large homogenous cell populations without increasing costs of potential therapy.^8^,^43^,^51^,^55^

At present, chondrocytes are obtained from the differentiation of multipotent and pluripotent cells by various protocols.^2^,^12^,^39^,^54^,^61^,^62^ These methods involve supplementation of the culture medium with specific growth factors, mainly bone morphogenetic proteins (BMPs) and transforming growth factor-beta (TGF-β) family members.^35^,^54^,^61^ These two protein families are responsible for chondrocyte maturation from mesenchymal cells during bone and cartilage development in the fetus. The results of numerous studies suggest that TGF-β_3_ has the strongest prochondrogenic properties compared with other growth factors.^4^,^20^,^34^,^37^ To the date, several improved protocols of *in vitro* chondrogenesis have been published. These protocols involve the induction of cartilage ECM components produced by specific physical and chemical factors i.e. hypoxic conditions of cell culture,^24^ three-dimensional systems,^30^,^60^ exposure to low pulsating ultrasound^6^,^41^ and mechanical forces caused by centrifugation or hydrostatic pressure.^31^,^46^ Moreover, research on biomaterials has demonstrated their usefulness in improving the differentiation process and *in vitro* propagation of cells.^16^,^27^,^48^,^53^,^58^

Various methods have been used to evaluate changes occurring within cells during the differentiation process. Molecular analysis of these changes involves detailed, expensive, complicated and time-consuming procedures linked to sophisticated bioinformatics analyses. However, some published protocols concerning the evaluation of protein expression are simple, specific and relatively inexpensive, for example, immunofluorescence (IF) labeling, which is commonly used and accessible in most laboratories.^38^ The IF technique allows researchers to evaluate levels of protein expression and monitor the number of biological processes.^32^,^47^ Flow cytometry enables quantitative and qualitative analysis of signal intensity and evaluation of percentage distribution of positively labeled cells within a sample population. However, this technique requires a large number of cells, the cost of the apparatus is relatively high and analysis must be performed by highly qualified staff.

Dynamic development of information technology has led to an increased availability of numerous applications for microscopic image analysis of various cells *in vitro*. Furthermore, the use of appropriate software, allows for rapid and cost-effective evaluation of the material, whilst reducing the volume of sample required.

The aim of this study was to test, the usefulness of semi-quantitative analysis of the signal intensity emitted following IF of labeled cells during the differentiation of hESCs into chondrocytes.

## Materials and Methods

### hESC Culture

Human embryonic stem cells (BGV01) were obtained from ATCC (VA, USA) and were cultured on mitomycin-C-treated mouse embryonic fibroblasts (MEFs, passage 3, Merck Millipore, Darmstadt, Germany) in hESC medium consisting of high-glucose DMEM/F12 (Merck Millipore) supplemented with 15% fetal bovine serum (FBS, Sigma-Aldrich, St. Louis, MO, USA), 5% knock-out serum replacement (KSR, Thermo Fisher Scientific Inc., Waltham, MA, USA), 1% penicillin–streptomycin (Sigma-Aldrich), 1 mM non-essential amino acid (NEAA, Merck Millipore), 0.2 mM 2-mercaptoethanol and 10 ng/mL basic fibroblast growth factor (bFGF, Merck Millipore). Cell cultures were maintained at 37 °C in a humidified atmosphere containing 5% CO_2_.

### Chondrogenic Differentiation of Embryoid Bodies

Embryoid bodies (EBs) were formed from previously trypsinized hESCs, which were seeded onto 96-well plates (BRAND inertGrade™, Wertheim, Germany) at 1000 cells/well. At day 7, the 2 of EBs were transferred into Matrigel™-coated 48-well plates (Matrigel™, Corning, NY, USA) and after 24 h the medium was switched to chondrogenic medium (ChM) (day 0). ChM was composed of DMEM/F12 (Merck Millipore), 10% FBS, 1 mM sodium pyruvate (Sigma-Aldrich), 10^−7^ M dexamethasone, 50 μM ascorbic acid, 50 μM l-proline, 1% penicillin–streptomycin (Sigma-Aldrich), 1% ITS^+^ premix (Corning, NY, USA) and 10 ng/mL TGF-β_3_ (Immunotools, Friesoythe, Germany). Culture was carried out for 20 days and the medium was changed every second day.

### Chondrogenic Differentiation of Monolayer Cells

The protocol for monolayer differentiation of hESCs into chondrocytes was established by Yang *et al*.,^61^ based on a simplified protocol by Oldershaw *et al*.^39^ Briefly, cells were differentiated for 2 weeks using various concentrations of growth factors: Wnt-3a (wingless-type MMTV integration site family, member 3A), activin-A (both from Sigma-Aldrich), growth/differentiation factor 5 (GDF-5, Immunotools), fibroblast growth factor 2 (FGF-2, Merck Millipore), BMP-4 (bone morphogenetic protein 4, Sigma-Aldrich) and follistatin (Peprotech, Rocky Hill, NJ, USA). 100,000 cells/well were seeded onto a six-well plate coated with Matrigel™. After cell attachment, the hESC medium was switched to ChM with cytokines. The time schedule and concentration of cytokines added at each day of differentiation are presented in Table S1 (supplementary data). Cells were cultured in serum-free ChM supplemented with 0.1% KSR (ThermoFisher Scientific Inc., Waltham, MA, USA).

### Indirect IF Staining

The 2 EBs, previously seeded onto 48-well plates, were fluorescently labeled at different time points of differentiation (days 0, 7, 14, and 21). Briefly, cells were washed with phosphate-buffered saline (PBS) and then fixed with cooled methanol for 20 min at −20 °C. Next, the cells were blocked with PBS containing 1% BSA (bovine serum albumin) and 0.5% Tween. Cell were then incubated for 1 h at 37 °C with primary antibodies diluted in PBS containing 1% BSA at previously optimized dilutions (a list of antibodies and their dilutions used for immunocytochemistry are enclosed in supplementary data, Table S2). After washing, a secondary antibody conjugated with Alexa-488 was added and the incubation was carried out for 1 h at 37 °C. After washing, the nuclei were stained with 4′,6-Diamidino-2-phenylindole dihydrochloride (DAPI) at 1:10,000 dilution (Sigma-Aldrich). Images were taken using a Leica Series fluorescent microscope at the same time of exposure and gain, which was optimized for each antibody. A feeder-free hESC cell line and primary human articular cartilage chondrocytes (ACC) were used as controls.

### The Evaluation of IF-Labeled Signal

**Figure 1:**
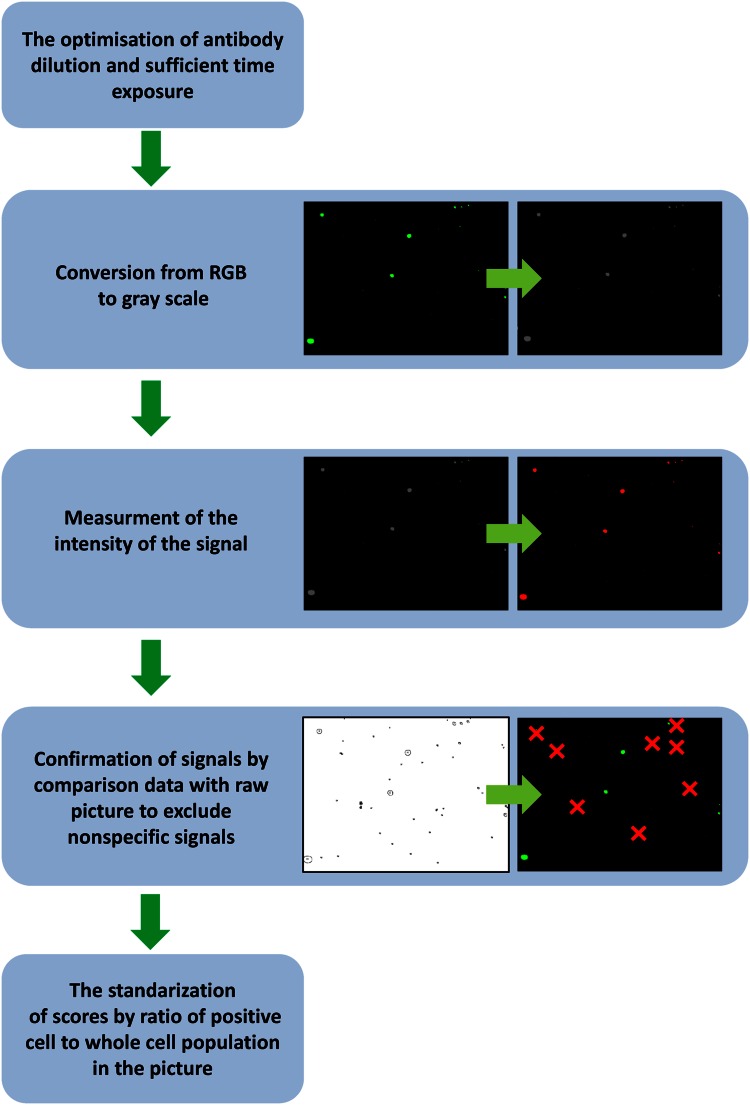
Step-by-step instructions for analysis of immunofluorescence images.

The intensity of the signals was evaluated (Fig. 1) using the bioinformatics programme ImageJ version 1.49j (developed by Wayne Rasband, NIH, USA). Images from the RGB format were converted into a 16-bit gray scale. Signal thresholds were set only in the area of the positive-labeled cells. The marked areas were described as a mean value of intensity of the pixels (ranging from 0 (dark) to 255 (white)). Because of non-specific signals such as non-specific binding or unwashed secondary antibodies, cell aggregate signals were excluded. After evaluation of the measurements, quantification of the positively stained cell population was conducted to standardize the scores. This was carried out by multiplication of the mean gray signals by the ratio of positively stained cells to the number of cells in the whole population. From the mean gray signals obtained from cell colonies, total mean gray values of the image were generated.

### Statistical Analysis

The comparison of mean gray intensity signals between two populations of differentiated cells and control populations (ACC, hESCs, and cells exposed to ChM) were evaluated by the unpaired tailed Student *t* test (QuickCalcs, GraphPad online statistical calculator; GraphPad, La Jolla, CA, USA). There were three levels of statistical significance: *p* < 0.05 (significant), *p* < 0.01 (highly significant), and *p* < 0.001 (extremely significant). SEM were calculated from the total mean gray intensity signals of gathered means from three pictures from wells of three independent cell cultures.

## Results

### Differentiation of hESC into Chondrocytes

The procedure of differentiation according to monolayer (2D) and EB (3D) differentiation protocols caused changes in morphology of cultured cells. During the differentiation process, cell elongation and an increased nucleus/cytoplasm ratio were observed (supplementary data, Fig. S1A and B). During the first 7 days of culture, growth factors, WNT-3a, BMP-4, activin-A, follistatin, and FGF-2 were added to the prochondrogenic medium, resulting in the development of mesoderm.^39^,^61^ Cells were then exposed to GDF-5, which is a member of the TGF-beta protein family, and after 14 days of culture chondrocyte-like cells were observed.

EBs are heterogeneous cell populations consisting of three germ layers. After attachment, the EB structures became flattened and cells of various shapes were detected. After 21 days of culture, chondrocyte-like cells were observed (Fig. S1, panel A). During differentiation at monolayer protocol, some cell detachment was observed, likely owing to decreased cell viability of some cells (Fig. S2). Previously, it was reported, that cells, which failure to differentiate into chondrocytes, die off.^39^,^61^ One of the reason was serum-free conditions or less enriched medium in EBs protocol. For hESCs propagated in richer medium, the poorer conditions could cause starvation stress. On the other hand, the increased amount of dead cells in EBs based protocol could be related to preparation of EBs by dissociation of hESCs to single cells, what decrease their viability. Another aspect is related to formation of necrotic area inside of EBs, which is related to hypoxia and lack of access to nutrients and growth factors in this area.^19^,^44^

### The Fluorescence Labeling of 2D and 3D Differentiated Cells

**Figure 2:**
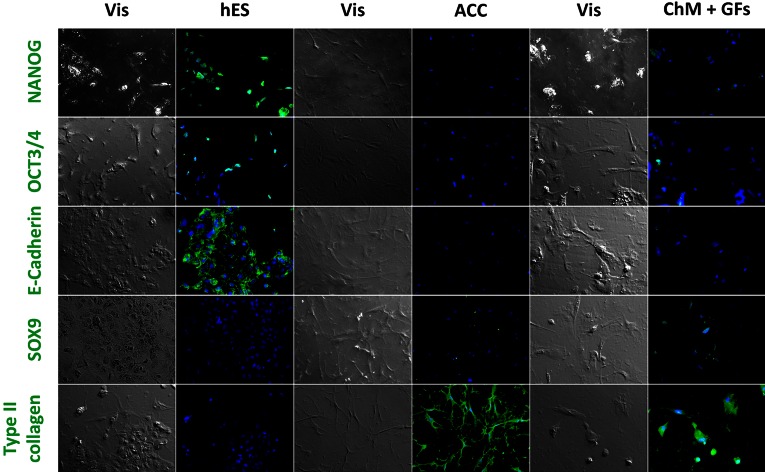
Immunofluorescence staining of differentiated hESCs into chondrocytes in a monolayer. Fluorescence-labeled cells for pluripotent (NANOG, OCT3/4, E-cadherin) and chondrocyte markers (SOX9, type II collagen) were used to evaluate the protocols for measurement of the mean gray intensity. Nuclei were stained using DAPI. Images were obtained using ×100 magnification.

After 2 weeks of differentiation, cells in monolayer culture were stained for both pluripotent (NANOG, OCT3/4, E-cadherin) and chondrogenic (type II collagen, SOX9) markers (Fig. 2). Bioimaging evaluation of the differentiated cells and comparison to the control hESC population, demonstrated a decreased number of positive-labeled cells with pluripotent markers, except for NANOG, which continued to be expressed, although at a low level. This was likely caused by the presence of FGF-2 in the medium, which is crucial for maintaining pluripotency.

**Figure 3:**
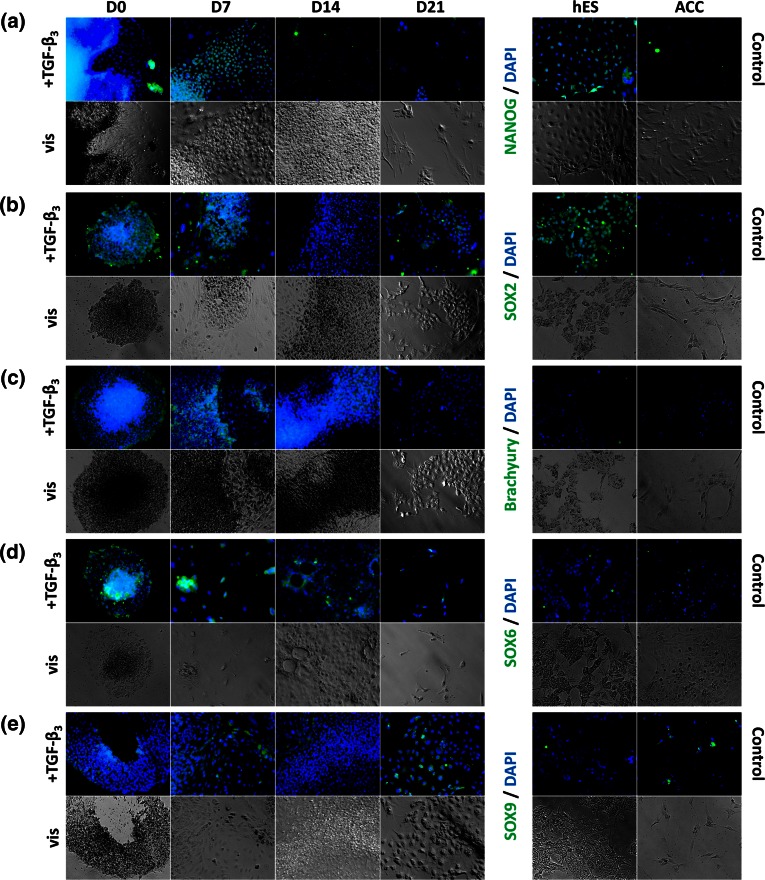
Immunofluorescence staining of various transcription factors during chondrogenic differentiation of EBs exposed to TGF-β_3_. EBs at different time points were stained for pluripotent markers (NANOG, SOX2), mesodermal marker (Brachyury) and prochondrogenic transcriptional factors (SOX6, SOX9). hESCs and ACCs were used as controls. Images were obtained using ×100 magnification.

**Figure 4:**
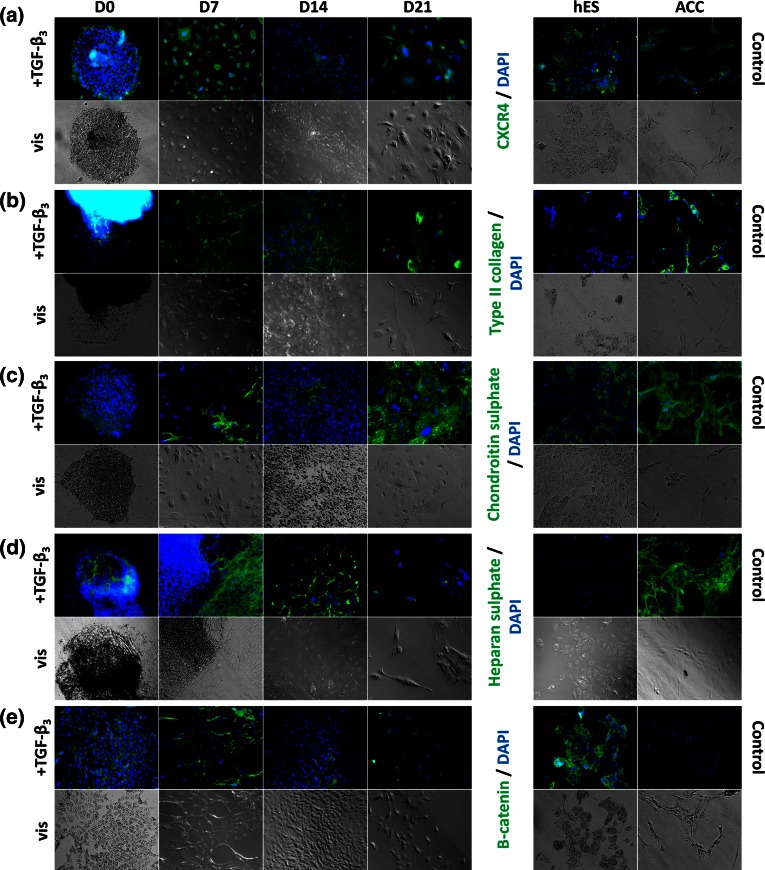
Immunofluorescence staining of ECM and surface proteins in EBs during chondrogenic differentiation. To confirm hESC differentiation into chondrocytes, cells were stained for cartilage markers collagen type II and Wnt signaling pathway activator, β-catenin. Cells were also labeled for chondroitin sulfate, heparan sulfate and CXCR4. To validate staining specificity, hESCs and ACCs were used as controls. Cells were observed and images were obtained using ×100 magnification.

Evaluation of EBs at the first time point (day 0) was difficult because of their flattened structure and central area rich in cells. This prevented positively labeled cells being distinguished from the general cell population. The condensed cells localized in the center of the flattened EBs were acknowledged as an artifact (Figs. 3a, 3c, and 3d—time point D0). As expected, pluripotent marker expression decreased throughout the differentiation process, whereby SOX2, NANOG signals were less intense (Figs. 3a and 3b). Brachyury, the mesodermal marker (Fig. 3c), was observed from day 0 onwards, which confirmed the presence of the mesodermal germ layer in the EBs. Prochondrogenic markers SOX6 and SOX9 (Figs. 3d and 3e) were clearly observed at day 21 in comparison to previous time points, whereas the expression of CXCR4 (chemokine (C-X-C motif) receptor 4) (Fig. 4a) appeared stable during differentiation. Immunofluorescence analysis of ECM components (type II collagen, chondroitin sulfate, heparan sulfate) (Figs. 4b–4d) at various time points during EB transition to chondrocytes, demonstrated increased expression of these markers. Moreover, primary chondrocytes and differentiated stem cells showed a similar extensive pattern of ECM marker expression. Detection of type II collagen at day 21 confirmed successful differentiation of the cells into chondrocytes. β-catenin (Fig. 4e) signal was less intense throughout the differentiation process.

### The Evaluation of Mean Gray Signal Intensity of IF-Labeled Cells

**Figure 5:**
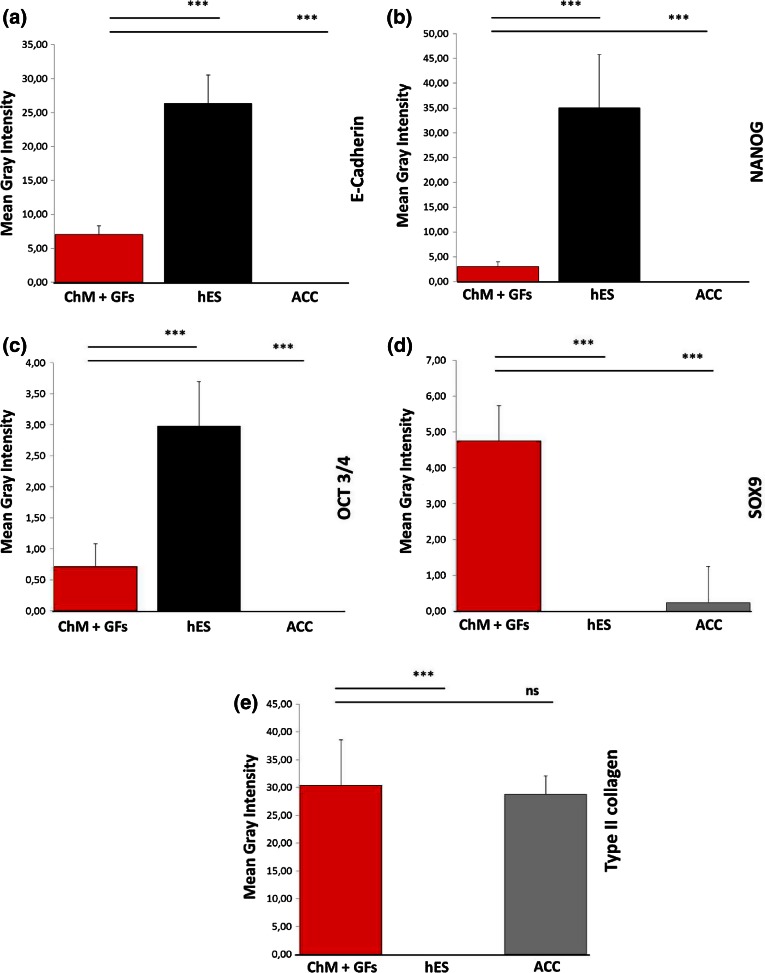
Analysis of fluorescence intensity level of differentiated hESCs into chondrocytes in a monolayer. Differences between the differentiated cell population (ChM + GFs) and controls (ACC; hESCs) were statistically significant (*p* < 0.001). Error bars represent standard error of the mean (SEM).

Analysis of monolayer (2D protocol) differentiated stained cells demonstrated extremely significant changes (*p* < 0.001) when compared with the control population (hECSs). Pluripotent marker (E-cadherin, OCT3/4, NANOG) (Figs. 5a–5c) expression levels were decreased below those of the control cells (NANOG, OCT3/4, E-cadherin). Moreover, after differentiation, chondrocyte markers such as collagen type II and its expression regulator, SOX9 displayed elevated values of signal intensity (Figs. 5d and 5e).

**Figure 6:**
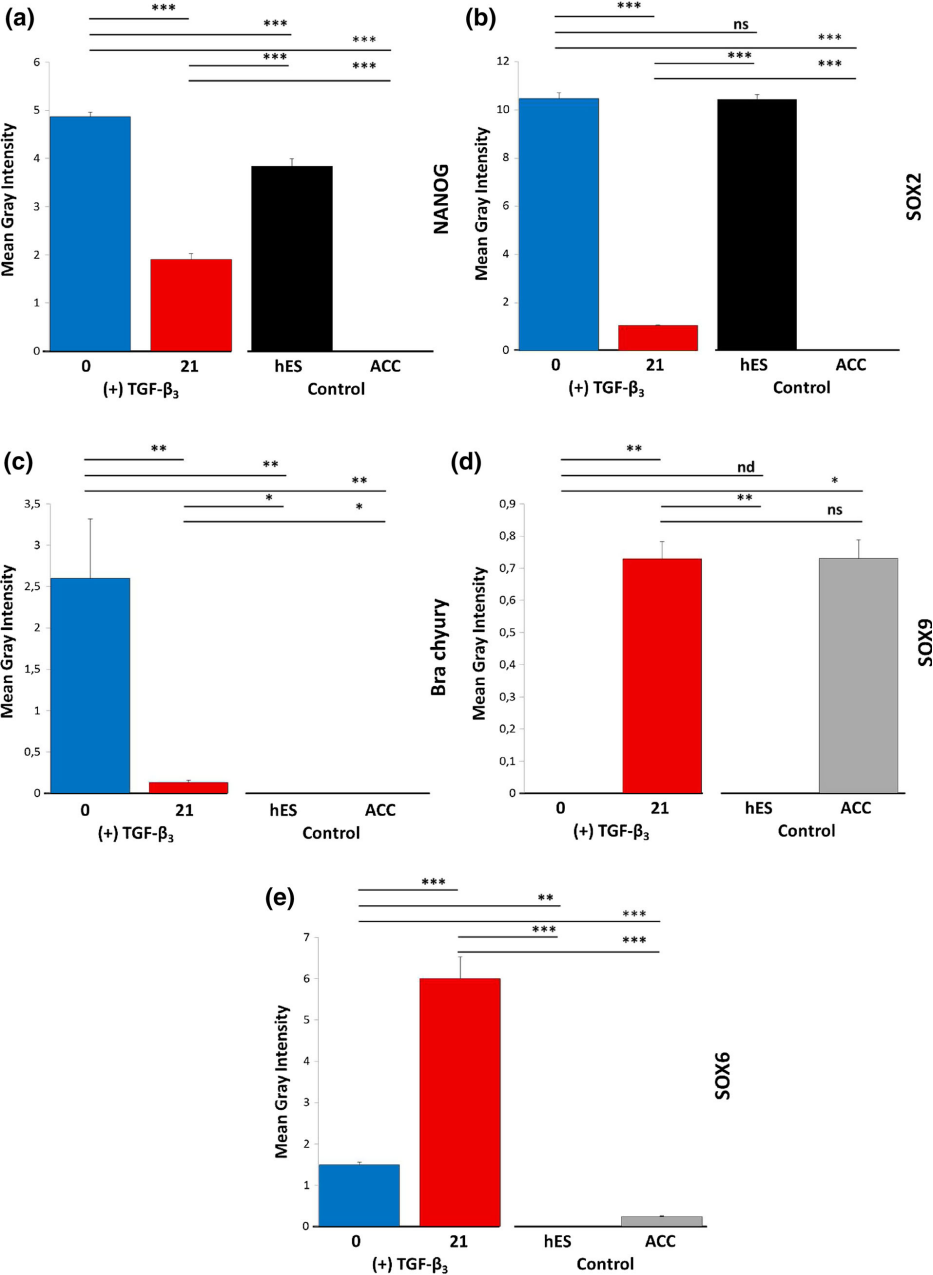
Analysis of mean gray signal intensity from cells stained for transcription factors during chondrogenesis. Changes between control cells (ACC, hES) and differentiated EBs ((+)TGF-β_3_) were observed. Statistically significant scores are highlighted (*=*p* < 0.05; **=*p* < 0.01; ***=*p* < 0.001; ns—not significant). Error bars represent SEM.

**Figure 7:**
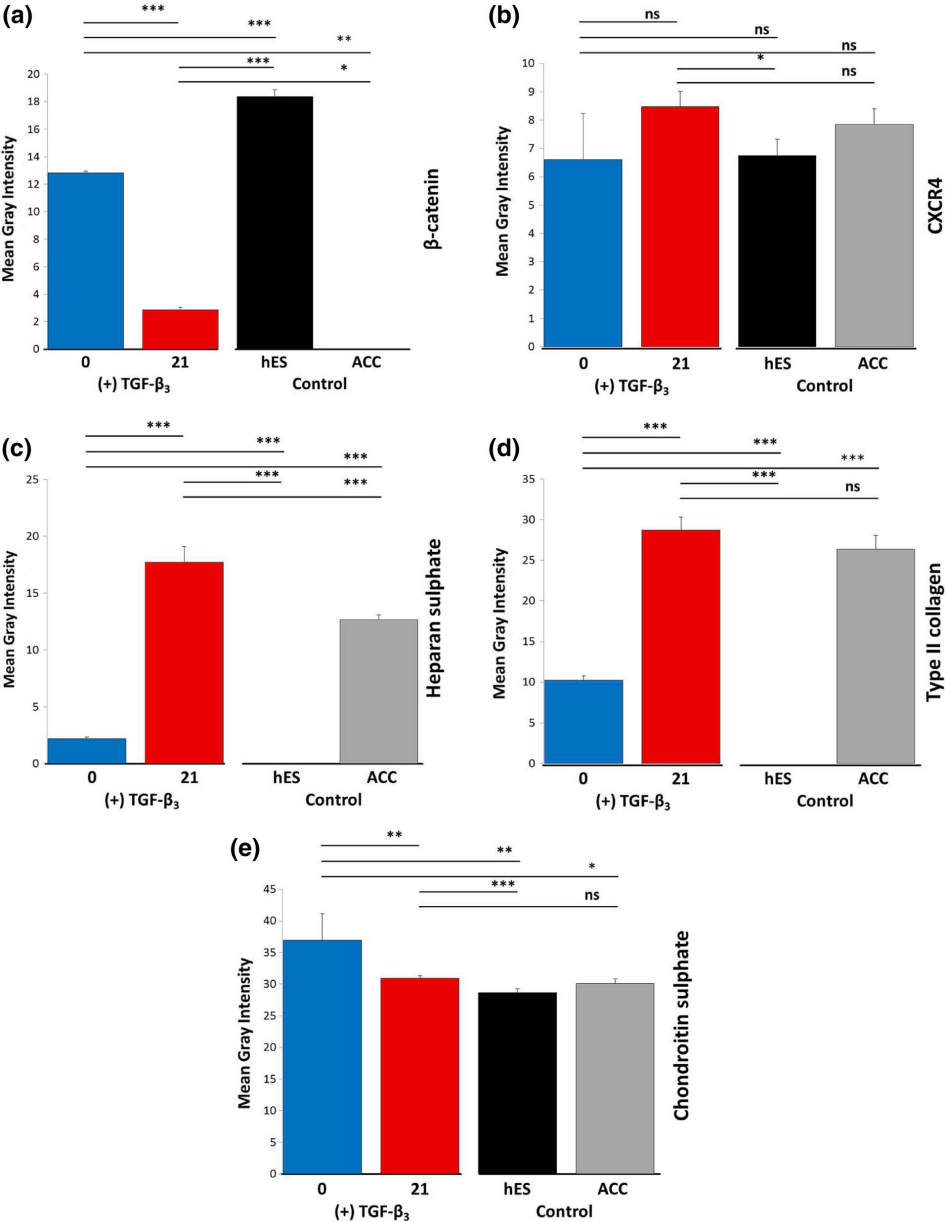
Analysis of mean gray signal intensity of immunolabeled surface proteins and ECM markers during chondrogenic differentiation of EBs. The mean gray intensity of EBs treated with TGF-β_3_ was compared with other cell populations signal emissions. Statistical significance is highlighted: *=*p* < 0.05; **=*p* < 0.01; ***=*p*<0.001; ns—non-significant; nd—no data to compare). Error bars represent SEM.

Between 0 and 21 days of EB (3D protocol) differentiation, major changes in fluorescence intensity levels were observed. Expression analysis of proteins responsible for the state pluripotency (NANOG) displayed an extremely significant difference (*p* < 0.001) when compared with the control cell population (Fig. 6a). Differences in the level of signal intensity for SOX2 between the differentiated cells and control ACCs and hESCs were also extremely significant (*p* < 0.001). Differences between individual time points were also extremely significant (*p* < 0.001) (Fig. 6b). Brachyury expression (Fig. 6c) tended to decrease, with the level of signal being statistically significant for differentiated cells in comparison to control cells (*p* < 0.05). Differences in Brachyury intensity signal between day 0 and other time points were also highly significant (*p* < 0.01). Expression of SOX9 (Fig. 6d) and SOX6 (Fig. 6e), represented by an increased level of gray signal intensity, was caused by transformation of cells into chondrocytes. There were no statistical significance differences of SOX9 intensity level at day 21 between differentiated cells and ACCs. Decreased signal intensity of β-catenin was observed during differentiation (Fig. 7a) with the difference between control cells and time points being very significant (*p* < 0.001; *p* < 0.05). Supplementation of chondrogenic medium with TGF-β_3_ did not significantly influence CXCR4 expression (Fig. 7b). After EB differentiation, an increase in the level of fluorescence intensity of cartilage-specific ECM components (heparan sulfate, type II collagen) was observed (Figs. 7c and 7d, *p* < 0.001). Chondroitin sulfate (Fig. 7e) signal intensity decreased between individual time points under exposure to growth factors, which could be explained by cell migration (*p* < 0.01). However, the level of fluorescence intensity of labeled chondroitin sulfate in ACCs and in the differentiated cells was almost equal.

## Discussion

Immunofluorescence labeling of cells is a fast and inexpensive method of monitoring biological processes. Converging the IF technique with contemporary bioinformatics approaches creates an opportunity to analyze and monitor not only the signal intensity but also the time course of changes observed within cells, whilst allowing statistical significance to be determined. The differentiation of hESCs into chondrocytes in both monolayer (2D) and EB (3D) resulted in heterogeneous populations of cells containing chondrocyte-like population, as supported by increased intensity of type II collagen and SOX9 signals after differentiation was complete. Expression of these proteins is usually used as a criterion for chondrogenic cell differentiation. In monolayer culture signal intensity of type II collagen was slightly higher than in differentiated cells compared with ACCs. These decreased values in ACCs could be correlated with loss of function, phenotype and increased proliferation during propagation of primary chondrocytes, what was described in several studies.^7^,^15^ An increased mean gray intensity was also observed for SOX9 in differentiated cells, where differences between the control and differentiated cells were notable. The increased level of intensity of SOX9 signal in the differentiated cells could also explain higher expression of type II collagen, because SOX9 acts as a transcription factor involved in regulation of COL2A1 expression. During differentiation, the pluripotency of hESCs was significantly decreased. Changes were observed for OCT3/4 and NANOG, well-known markers of pluripotency. These results were substantiated by very low expression of E-cadherin in comparison to the controls (*p* < 0.001). This adhesive protein plays an important role in maintaining the pluripotency state, as clearly shown for induced murine pluripotent stem cells.^45^

Supplementation of prochondrogenic medium with TGF-β_3_ resulted in increased production of ECM components following 3D differentiation culture. Type II collagen was present in EBs from day 0, which is not surprising because EBs constitute a heterogeneous group of cells, whilst at day 21 signal intensity was significantly increased. The presence of SOX6, but not SOX9, was detected at day 0. Transcription factors SOX6, SOX9, and L-SOX5 are crucial regulators of expression of ACAN, which is responsible for the synthesis of aggrecans and genes regulated by collagens type II, IX, and XI.^15^,^62^

From the beginning of differentiation, chondroitin sulfate was expressed in the differentiated cells and control hESCs and ACCs. Expression of chondroitin sulfate in hESCs has an important function during early embryo development, alongside E-cadherin, to maintain the balance between self-renewal and differentiation.^21^ Moreover, the pattern of signal and surface coverage of this marker changed during differentiation. After differentiation, the ECM area was larger and less condensed than in the hESCs and EBs at day 0, and resembled the ACC signal pattern. Signal intensity decreased during differentiation and at the end of the time course, differentiated cells gained a similar level of intensity to ACCs. With respect to the ECM component, heparan sulfate expression was increased during differentiation. At day 21, the pattern of ECM distribution in EBs, which had undergone differentiation, was dissimilar to that of ACCs, but the level of signal intensity of the labeled structures was increased in comparison to day 0 and to ACCs. To explain this occurrence, it should be noted that during differentiation cell proliferation increased and after day 14 cell dissociation was necessary. This caused reduced cell attachment to the well surface. However, the signal intensity was higher because of labeled intracellular compartments containing heparan, which were more condensed than externalized and distributed from ECM presented in ACC culture. Surprisingly, this phenomena of internalization in other ECM components were not observed. We suppose that, it could be resulted by the lack of an appropriate cell number sufficient to co-stimulatory secretion of heparan sulfate into outer membrane due to its important role of cell–cell interactions by binding some receptors, cytokines and integrins.^49^

From the beginning of the differentiation process, the mesoderm marker Brachyury, which is encoded by the T gene, was expressed. This was expected as EBs spontaneously differentiate into cells of all three germ layers. During embryonic development, the condensed mesenchymal cells, stimulated by the TGF-β family of proteins, create a cartilage nodule, which during further differentiation, develops into limbs and joints.^40^ This is why many protocols for chondrocyte differentiation use mesenchymal stem cell populations. The lower fluorescence intensity signal of Brachyury, even after completion of the differentiation protocol, was probably caused by transition of mesodermal cells into chondrocytes.

During chondrogenic differentiation of EBs exposed to TGF-β_3_, a lower signal intensity of β-catenin was observed at day 21 in comparison to day 0. The main cause of this decreased signal is connected with interactions between SOX9 and β-catenin resulting in decreased pluripotency.^1^ β-Catenin is present in mesenchymal cells but during mesenchymal cell condensation and differentiation towards chondrocytes its expression is down-regulated.^14^

After differentiation was completed, the signal intensity of prochondrogenic markers was increased, whilst expression of the pluripotent transcription factors, SOX2 and NANOG, was decreased, indicating loss of pluripotency during differentiation of EB cells into chondrocyte-like population. In accordance with previously elaborated protocols, type II collagen positive cells were generated. Moreover, the expression of transcription factors characteristic for chondrocytes (SOX6, SOX9) was confirmed. Similar expression profiles during chondrocyte differentiation (down-regulation of pluripotency markers and upregulation of prochondrogenic markers) were observed in various protocols.^18^,^26^,^36^,^39^ To quantify the efficiency of in vitro chondrogenesis, generally RT-qPCR expression analysis is used. Based on this analysis, the expression of pluripotency markers such as NANOG, OCT3/4, E-cadherin (CDH1), SOX2, and mesodermal marker T (Brachyury) was decreased in various differentiation protocols.^26^,^39^ CXCR4 gene expression level during differentiation protocol performed by Oldershaws’ group, were not statistically changed. What more, the gene expression of prochondrogenic markers were increased in the end of hESc differentiation for ACAN (upregulated 2.5-fold), SOX6 (upregulated 3.6-fold), SOX9 (upregulated 5-fold) and COL2A1 (upregulated 370-fold).^39^ In our study to analyze the differentiation efficacy, we took advantage of immunofluorescence as an optimal tool to analyze protein expression and location. Analysis of protein, as a final product of gene expression, allows to see the real functional products of cells, because it is known, that not all of the transcript can undergo translation.^29^

In our study, we presented the commonly used method of indirect IF with slight analysis modifications, which allows to semi-quantification of the results by taking under consideration the mean intensity of signals in whole cell population on the image. Our quantitative analysis correlates with recently published data concerning chondrogenic differentiation of pluripotent stem cells.^9^,^26^,^39^ The availability of free bioinformatics tools such as ImageJ, enables to easily analyze data and extract additional information from pictures by modification of program for own purpose. The indirect immunofluorescence is suitable technique to detect various proteins of cytoskeleton, ECM, which enables the visualization of the phenotype and structure of cells. However, in many published date researcher mostly used indirect IF only to confirm the presence of protein without further analysis and quantification.^22^,^28^,^32^,^39^ A few studies over improvements of articular cartilage functions or differentiation of bone marrow cells towards chondrocytes have used evaluation of IF images to count percentage of positively stained cells or measure the mean intensity by manual drawing area of the signal.^3^,^59^ In presented study by using threshold of RGB converted images into 16-bit picture, we can describe the exact specific-signal area and count mean gray value. Additionally, the standardization of those results by ratio of positively stained cells in our case enabled to better description of signal distribution in whole cell population.

It is worth mentioning, that our method is suitable only for cells, which are in monolayer cell culture, because of the 3D structure such as EBs enable to evaluate and interpret properly large mass of cells (such as in day 0 in EBs differentiation protocol). For 3D objects it seems better to use confocal microscopy or epi-fluorescence microscopy. The results obtained in this study confirm the usefulness of IF analysis to monitor the level of signal intensities of distinct cell populations during chondrogenesis and allows to track the changes of hES during differentiation to chondrocyte-like cells.

## Electronic supplementary material

Below is the link to the electronic supplementary material.

Supplementary material 1 (PDF 874 kb)
