# Supplementary material for: Bioimaging: An Useful Tool to Monitor Differentiation of Human Embryonic Stem Cells into Chondrocytes
Source: Ann Biomed Eng. 2015 Sep 9;44:1845–59. doi: 10.1007/s10439-015-1443-z (PMC4837225; doi:10.1007/s10439-015-1443-z)
Supplement: Supplementary file 1 — Supplementary material 1 (PDF 874 kb) [file 10439_2015_1443_MOESM1_ESM.pdf]

Annals of Biomedical Engineering

Research Article:

Bioimaging — a semi-quantitative tool to evaluate differentiation of human embryonic stem cells into chondrocytes

Wiktoria M. Suchorska [1]

Michał S. Lach [1, 2*]

Magdalena Richter [3]

Jacek Kaczmarczyk [3]

Tomasz Trzeciak [3]

[1] Radiobiology Lab, Greater Poland Cancer Centre, Garbary 16 Street, Poznan 61-866, Poland

[2] Postgraduate School of Molecular Medicine, Warsaw University of Medical Sciences, Warsaw, Poland

[3] Department of Orthopaedics and Traumatology, Poznan University of Medical Sciences, Poznan, Poland

* Corresponding author: Michal Lach, Garbary 16 Street, Poznan 61-866, Poland,

Phone number: +48 (61) 8550-474

e-mail: michal.lach@wco.pl, lach.michal89@gmail.com,

Table S1. The schedule of supplementing chondrogenic medium with growth factors during the differentiation process (Yang et. al 2012).

DAY	WNT3a	Activin-A	FGF2	BMP4	Follistatin	GDF5
**1**	25	50				
**2**	25	25	20			
**3**	25	10	20	40		
**4**			20	40	100	
**5**			20	40	100	
**6**			20	40	100	
**7**			20	40	100	
**8**			20	40		
**9**						40
**10**						40
**11**						40
**12**						40
**13**						40

Table S2. Primary and secondary antibodies and their dilutions used for immunocytochemistry protocols.

Name	Company	Dilution
**Primary antibody**		
NANOG	Abcam	1:50
NANOG	Thermo Fisher	1:50
OCT-3/4	BD Pharmigen	1:50
SOX2	BD Pharmigen	1:50
SOX6	Abcam	1:50
SOX9	Abcam	1:50
Collagen type II	Abcam	1:100
B-catenin	BD Pharmigen	1:100
CXCR4	Abcam	1:100
BRACHYURY	Abcam	1:50
Chondroitin sulphate	Abcam	1:100
Heparan sulphate	Abcam	1:100
E-cadherin	Abcam	1:100
**Secondary antibody**		
Anti-rabbit IgG conjugated with AlexaFluor-488	Jackson ImmunoResearch	1:500
Anti-mouse IgG conjugated with AlexaFluor-488	Jackson ImmunoResearch	1:500

Anti-mouse IgM conjugated with AlexaFluor-488	Jackson ImmunoResearch	1:500

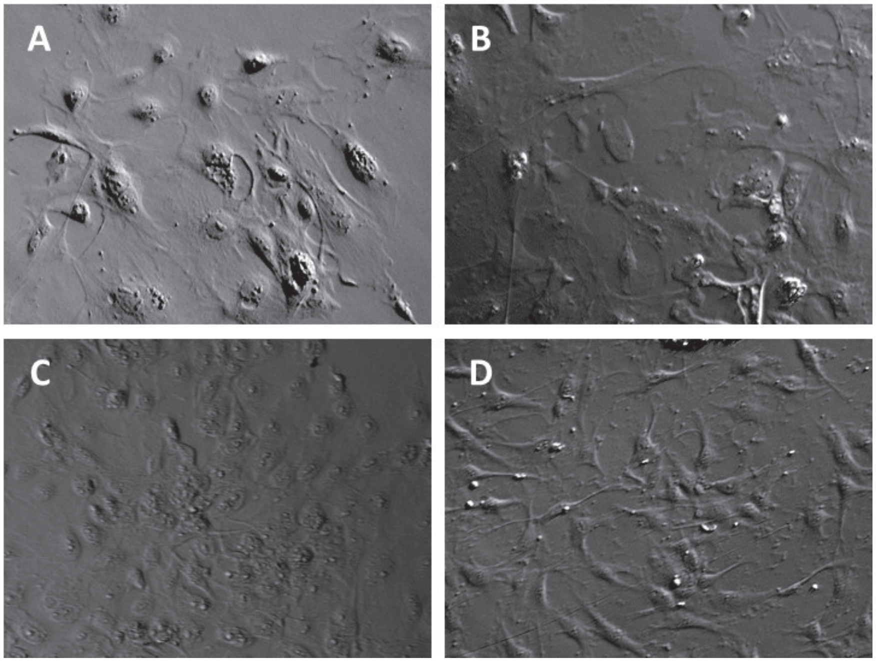

Figure S1. Differentiation protocols for obtaining chondrocytes from monolayer and EBs. Cells obtained from EBs supplemented with TGF-β_3 after 21 days (A) and exposure to various growth factors in monolayer cell culture after 14 days (B). As a control, human embryonic cells without MEFs were used (C) and chondrocytes obtained from waste material (D). Images were obtained using 100 × magnification.

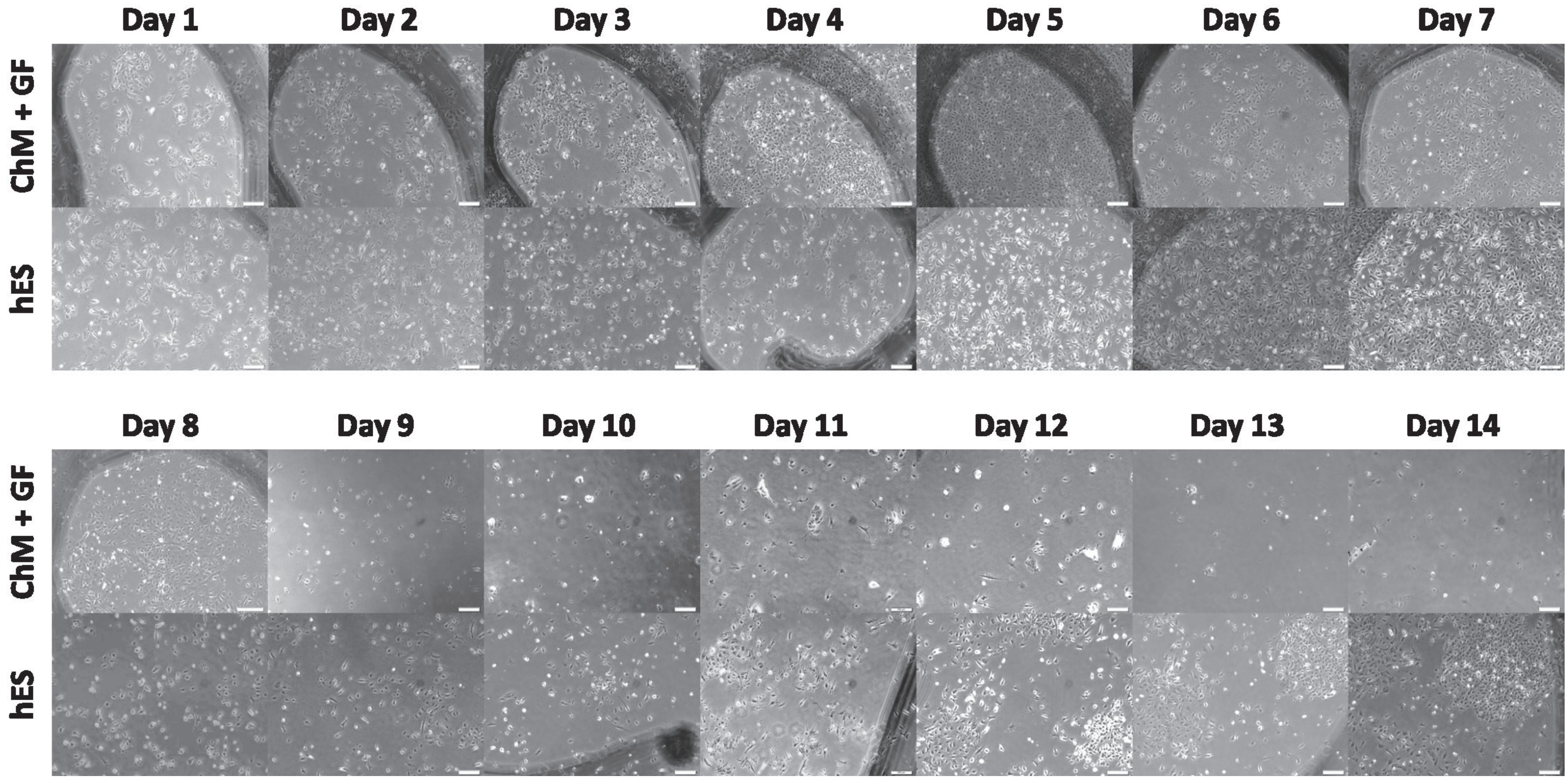

Figure S2. The observation of differentiation of hES cells into chondrocytes by monolayer protocol (14 days). During culture of ChM + GF cells, the growth of cells were increased what resulted in passage at 5th and 8th. On the following days it was observed decreasing proliferation of cells. What more, cells which did not undergo differentiation were detaching from surface of culture dish. As a control cell population hES were used. The white bar indicates 200µm. Pictures were taken under 100x magnification.
